# The Subtle Violence of the Caregiving Crisis

**Published:** 2008-06-15

**Authors:** Jayne Cagle

**Affiliations:** Chattanooga, Tennessee

## To the Editor:

Thank you for printing Rosalynn Carter's editorial on addressing the caregiving crisis (1), and bless Mrs Carter for helping to draw attention to this crisis. My husband Ben and I recently buried Ben's 85-year-old father, Berton, after spending 3½ years as his primary caregivers. Berton was a member of the "Greatest Generation"; he tirelessly served his country and his community throughout his life. His wife, Ruby, served in Admiral Nimitz's secretarial pool at Pearl Harbor, where Berton and Ruby met and married in 1945.

As caregivers, Ben was responsible for the "big picture," while I took care of details. Ruby was available on a daily basis and provided care and nurturing to the best of her abilities. Berton was quite active right up until the end.

Unfortunately, during Berton's final days, Ben and I felt that some members of the health care team never considered us to be full partners in Berton's caregiving. Some health care facilities seemed complacent in terms of standards of caregiving. On the basis of our recent experience, I agree with Mrs Carter: it **is** time for us to build communities of care. We owe it to our aging parents and grandparents, and we owe it to ourselves.

Ben and I look forward to visiting and learning more about the Carter Center. We have always thought highly of President and Mrs Carter, and reading online about the Carter Center prompts me to consider the possibility of making a difference by helping Mrs Carter improve caregiving in this nation. Not to do so would be turning my back on the subtle violence of this crisis.
